# Lymph Node Follicle‐Targeting STING Agonist Nanoshells Enable Single‐Shot M2e Vaccination for Broad and Durable Influenza Protection

**DOI:** 10.1002/advs.202206521

**Published:** 2023-04-24

**Authors:** Hsiao‐Han Tsai, Ping‐Han Huang, Leon CW Lin, Bing‐Yu Yao, Wan‐Ting Liao, Chen‐Hsueh Pai, Yu‐Han Liu, Hui‐Wen Chen, Che‐Ming J. Hu

**Affiliations:** ^1^ Institute of Biomedical Sciences Academia Sinica Taipei 115 Taiwan; ^2^ Taiwan International Graduate Program in Molecular Medicine National Yang Ming Chiao Tung University and Academia Sinica Taipei 112 Taiwan; ^3^ Department of Veterinary Medicine National Taiwan University Taipei 10617 Taiwan; ^4^ Biomedical Translation Research Center Academia Sinica Taipei 115 Taiwan; ^5^ Center of Applied Nanomedicine National Cheng Kung University Tainan 70101 Taiwan

**Keywords:** follicular dendritic cells, germinal center, lymph node follicle targeting, matrix protein 2 ectodomain antigen, nanoshell, stimulator of interferon genes agonist, universal influenza vaccine

## Abstract

The highly conserved matrix protein 2 ectodomain (M2e) of influenza viruses presents a compelling vaccine antigen candidate for stemming the pandemic threat of the mutation‐prone pathogen, yet the low immunogenicity of the diminutive M2e peptide renders vaccine development challenging. A highly potent M2e nanoshell vaccine that confers broad and durable influenza protectivity under a single vaccination is shown. Prepared via asymmetric ionic stabilization for nanoscopic curvature formation, polymeric nanoshells co‐encapsulating high densities of M2e peptides and stimulator of interferon genes (STING) agonists are prepared. Robust and long‐lasting protectivity against heterotypic influenza viruses is achieved with a single administration of the M2e nanoshells in mice. Mechanistically, molecular adjuvancy by the STING agonist and nanoshell‐mediated prolongation of M2e antigen exposure in the lymph node follicles synergistically contribute to the heightened anti‐M2e humoral responses. STING agonist‐triggered T cell helper functions and extended residence of M2e peptides in the follicular dendritic cell network provide a favorable microenvironment that induces Th1‐biased antibody production against the diminutive antigen. These findings highlight a versatile nanoparticulate design that leverages innate immune pathways for enhancing the immunogenicity of weak immunogens. The single‐shot nanovaccine further provides a translationally viable platform for pandemic preparedness.

## Graphical abstract

A nanoshell vaccine capable of retaining peptide antigens in the follicular dendritic cell network enables prolonged antigen exposure for B cell engagement. In conjunction with STING agonists to promote T cell help and germinal center formation, a universal influenza nanovaccine is developed to elevate humoral responses against a conserved influenza peptide antigen, enabling single‐shot protection against heterotypic influenza viruses.

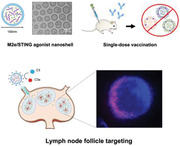

## 1 Introduction

Influenza poses a persistent public health concern with annual epidemics incurring serious morbidity and mortality.^[^ ^1^ ^]^ As a seasonal event, influenza is estimated to cause 3 to 5 million cases of severe illness with up to 650 000 respiratory deaths every year. Vaccination efforts against the mutation‐prone virus are encumbered by the viruses' tendency to undergo antigenic shift and antigenic drift, where reassortment of viral genes and point mutations at the receptor binding proteins can impair protectivity from pre‐existing antibodies.^[^ ^2^ ^]^ As such, current hemagglutinin‐based vaccines require annual updates,^[^ ^3^ ^]^ and they are susceptible to low protectivity in cases where the vaccines do not match with the circulating virus strains.^[^ ^4^ ^]^ Toward addressing the shortcomings of current influenza vaccines, a variety of vaccine designs based on both B cell‐ and T cell‐based immunogens have been proposed for universal influenza vaccination.^[^ ^5^ ^]^ Among them, peptide antigen derived from the extracellular domain of the ion channel membrane matrix protein 2 (M2e) of influenza viruses presents a unique target as it mediates antibody‐dependent cellular cytotoxicity (ADCC), which can eradicate M2e‐presenting infected cells prior to virus release and propagation. The highly conserved nature of M2e across human seasonal influenza A viruses makes it an attractive candidate for universal influenza vaccine development.^[^ ^6^ ^]^ However, the diminutive peptide antigen's low immunogenicity presents a major barrier against its clinical translation, with prior clinical trials of M2e vaccine candidates yielding unsatisfactory and declining humoral responses despite repeated inoculations.^[^ ^7^ ^]^ More recently, emerging recombinant protein strategies, carrier technologies, and immune‐stimulating adjuvants have renewed enthusiasm toward M2e‐based universal influenza vaccines.^[^ ^8^, ^9^, ^10^ ^]^ Yet despite advances in antigen and vaccine designs, multi‐dose regimens remain necessary to boost the peptide's immunogenicity, and the M2e antigen is frequently relegated to a complementary role to other immunogens owing to its partial protectivity.^[^ ^10^ ^]^ As an abbreviated vaccine regimen, as well as formulation simplicity, have immense value in improving vaccination logistics and public health strategies, a single‐shot M2e peptide vaccine capable of broad‐spectrum and durable influenza protection remains a highly desirable yet elusive goal. A nanoparticulate vaccine strategy is herein devised to enhance antigen availability and T cell help in the lymph node follicles for boosting M2e antigen immunogenicity.

**Figure 1:**
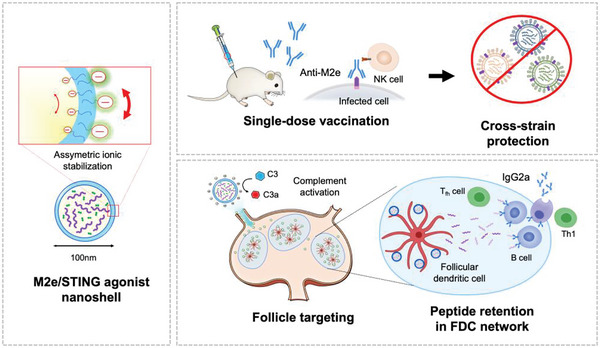
Design (left), application (top right), and mechanism (bottom right) of a single‐shot M2e‐based influenza vaccine for broad influenza protection.

To construct an M2e nanovaccine with high‐density co‐encapsulation of the peptide antigens and immunologic adjuvants, we demonstrate an asymmetric ionic stabilization strategy for the preparation of polymeric nanoshells. The stabilization strategy mimics the asymmetric stabilization mechanism behind nanoscopic biological vesiculation,^[^ ^11^ ^]^ which overcomes the energetic barriers in nanoscale curvature formations, thus preventing nanoemulsion collapse and enabling consistent preparation of antigen‐loaded nanocapsules in the absence of surfactants and stabilizers. A putative stimulator of interferon genes (STING) agonist cyclic‐di GMP (cdGMP) is applied as the adjuvant of choice due to its role in inducing type I interferons, a cytokine conducive to Th1‐biased production.^[^ ^12^ ^]^ The particular antibody isotype is required for anti‐M2e‐based ADCC against influenza‐infected cells.^[^ ^13^ ^]^ Upon assessment of the M2e nanoshell (NS(M2e+cdGMP)), we show that the nanovaccine is highly effective in promoting IFN*γ* ^+^ Type 1 helper T cells (Th1) induction, germinal center formation, and Th1‐skewed anti‐M2e production, and a single M2e nanoshell inoculation conferred complete and long‐lasting protection against lethal influenza challenge, enabling resolution of viral titers and prevention of lung immunopathology and tissue injuries. Full protectivity against heterosubtypic influenza challenges was also achieved under the single‐shot regimen. Intrigued by the extraordinary humoral responses of the M2e nanoshells, which have a distinctive design from conventional vaccines that display immunogens on carrier surfaces for B cell engagement, we interrogated how the nanoshells modulate the distribution of encapsulated antigens. The nanoshells were observed to shuttle the M2e peptides to the follicular dendritic cell (FDC) network in a complement‐dependent fashion, allowing for sustained peptide exposure in the B cell follicles for antibody induction. Notably, the incorporation of commonly adopted polyethylene glycol (PEG) surface coating on the M2e nanovaccines abrogated peptide retention in the FDC network and reduced antibody induction, highlighting the importance of surfactant‐free nanoshell design for maximizing follicle targeting (**Figure** **1**). The study presents a highly effective and translationally viable universal influenza vaccine candidate and introduces an FDC‐targeting nanoparticle design for improving antigen immunogenicity.

## 2 Results

### 2.1 Asymmetric Ionic Stabilization Enables High‐Density Co‐Encapsulation of M2e Peptides and STING Agonists in Polymeric Nanoshells

**Figure 2:**
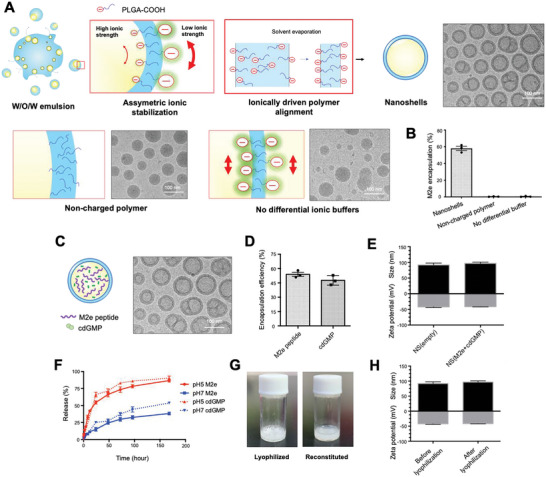
Preparation and characterization of M2e nanoshell vaccine. A) Schematics for the asymmetrically stabilized nanoemulsion process for nanoshell vaccine preparation and cryoEM images of polymeric nanoshells. The absence of charged polymer or differential ionic buffers led to emulsion collapse. B) Quantification of M2e peptide encapsulation following different emulsion processes for nanoparticle preparation. C) CryoEM visualization of M2e nanoshell vaccine co‐encapsulating M2e peptide antigens and cdGMP. Scale bars = 100 nm. D) Encapsulation efficiency of M2e peptides and cdGMP by the M2e nanoshell vaccine. E) The size and zeta potential of empty nanoshells (NS(empty)) and M2e nanoshell vaccine (NS(M2e+cdGMP)) were measured by dynamic light scattering. F) Release kinetics of M2e peptides and cdGMP from nanoshell vaccines at pH 5 and pH 7. G) Images of M2e nanoshell vaccine following lyophilization and reconstitution. H) The size and zeta potential of M2e nanoshells as measured by DLS show comparable physicochemical properties before and after lyophilization.

We have previously shown that the adoption of short poly(lactic‐co‐glycolic acid) (PLGA) polymers with low viscosity can reduce interfacial tension during double emulsion for hollow nanoparticle construction.^[^ ^14^ ^]^ To facilitate high‐density co‐encapsulation of M2e peptide antigens and hydrophilic cdGMP, we further improved the stabilization strategy with inspirations from the budding and vesiculation mechanisms of biological nanovesicles, which exploit asymmetric forces across the endoplasmic and exoplasmic surfaces for membrane curvature stabilization.^[^ ^15^ ^]^ We employed an asymmetric ionic stabilization strategy for water‐in‐oil‐in‐water (W/O/W) emulsion, which subjects anion‐bearing polymers (carboxyl‐terminated PLGA (PLGA‐COOH)) to high ionic strength buffer in the inner aqueous phase and low ionic strength buffer in the outer aqueous phase. The differential buffer contents impose asymmetric ionic screening on the exposed anions. With the inner buffer exerting a higher ionic screening effect as compared to the outer buffer, the anions at the endoplasmic interface experience reduced long‐range electrostatic forces and repulsion as compared to the exoplasmic side, thus generating an asymmetric strain that bends toward the encapsulant phase and facilitates nanocapsule stabilization. The highly polar carboxyl group of the amphiphilic polymer also facilitates polarity‐driven polymer alignment during the double solvent evaporation, giving rise to uniform shell formation upon polymer hardening. The simple yet intricate emulsion protocol led to the formation of monodisperse, surfactant‐free nanoshells that readily partitioned desired encapsulants in its inner aqueous core (**Figure** **2**A,B), and M2e peptide encapsulation with the nanoshells showed a consistent efficiency of ≈55% with input peptide concentrations ranging from 2.5 to 40 mg mL^−1^ (Figure S1, Supporting Information). Notably, double emulsions in the absence of anionic polymers or differential buffers across the inner and outer aqueous phases led to the collapse of the capsule structure and poor antigen encapsulation as the nanoparticulates assumed a more energetically favorable solid conformation with reduced interfacial areas (Figure 2A,B). Control of the ionic strength of the inner aqueous phase favored negative curvature formation around the encapsulants during the emulsion process, thus providing a robust and versatile approach for modular cargo encapsulation.

M2e nanoshell vaccines were prepared with an inner aqueous phase solution containing 40 mg mL^−1^ M2e peptides and 5 mg mL^−1^ cdGMP, and, in spite of the high encapsulant content, the resulting nanoshells retained well‐defined core–shell structures (Figure 2C), high encapsulation efficiencies (Figure 2D), and identical physicochemical properties as empty nanoshells (Figure 2E). The M2e nanoshells had a unimodal size distribution with an average diameter of 98.7 nm and a zeta potential of −42.7 mV. Antigen, adjuvant, and nanoparticle quantification by BCA assay, high‐performance liquid chromatography, and nanoparticle tracking analysis showed that each nanoshell contained ≈7600 peptide antigens and 3000 cdGMP molecules (Figure S2, Supporting Information). The acid‐labile biodegradability of the nanoshell bestows a pH‐responsive release kinetics for the encapsulated peptide and adjuvant. At physiological pH (≈pH 7.4), M2e nanoshells showed sustained M2e peptide and cdGMP release profiles with ≈50% of the encapsulants being retained in the particles by day 7. At pH 5, the accelerated ester hydrolysis of the PLGA shell sped up the cargo release with 54.5% of the peptides and 66% of cdGMP escaping within 1 day (Figure 2F). The M2e nanoshells were further demonstrated to be highly stable following lyophilization. Upon reconstitution following 1 month of storage at room temperature in powdered form, the NS(M2e+cdGMP) retained equivalent size, surface charge, and antigen encapsulation to that of freshly prepared samples (Figure 2G,H and Figure S3, Supporting Information). These characterizations highlight multiple desirable features of M2e nanoshells for clinical translation including ease of preparation, biocompatibility, and storability.

### 2.2 Single‐Dose M2e STING Agonist Nanoshell Induces Robust Anti‐M2e IgG2a for Antibody‐Dependent Cell‐Mediated Cytotoxicity

**Figure 3:**
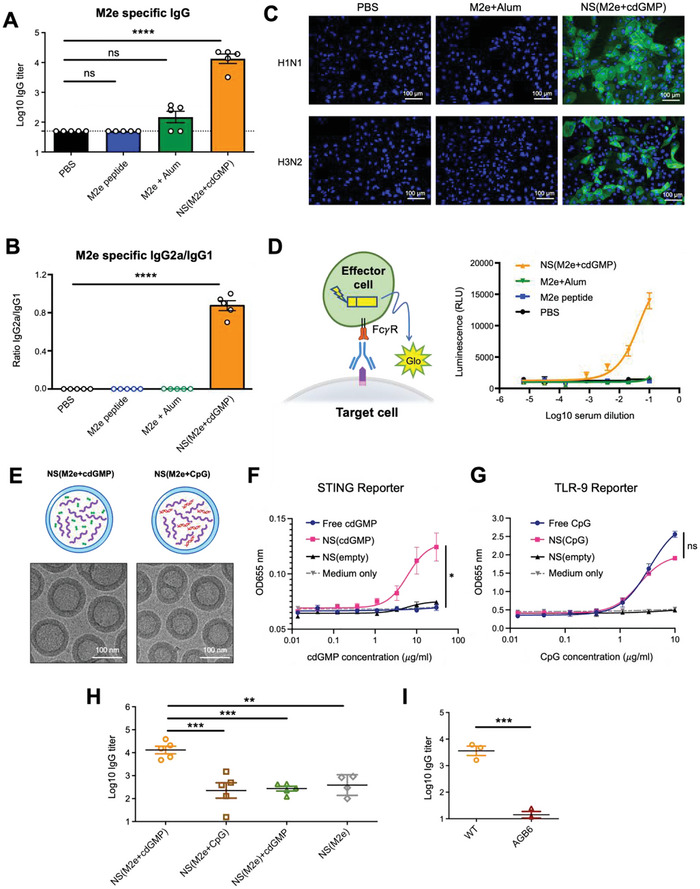
Anti‐M2e induction and ADCC activity following M2e nanoshell vaccine inoculation in mice. A) M2e‐specific IgG titers following a single shot immunization with PBS, M2e peptide, Alum‐adjuvanted M2e peptides, and M2e nanoshell vaccine in mice. B) M2e‐specific IgG2a to IgG1 titer ratios in immunized Balb/C mice on day 35 post‐vaccination. Error bars represent mean ± SEM (*N* = 5). C) Acetone‐fixed MDCK cells with heterotypic influenza infections for evaluating antibody binding to cell‐bound M2e by anti‐M2e from mice serum. Immunofluorescence assays were performed using mice serum derived on day 42. Nuclei were stained with DAPI. H1N1: A/Puerto Rico/8/1934 (H1N1); H3N2: A/HKx31 (H3N2). Scale bars = 100 µm. D) ADCC surrogate assay with mice serum derived on day 42 post‐vaccination against H1N1‐infected MDCK cells. Data are presented as mean ± SEM. (*N* = 3). E) CryoEM images showing the morphology of nanoshells encapsulating the combinations of either M2e + cdGMP or M2e + CpG‐ODN 1826. F) Assessment of human STING activation by SEAP reporter cells with free cdGMP, NS(cdGMP), or empty NS following incubation for 24 h. G) Assessment of human TLR9 activation by SEAP reporter cells with free CpG‐ODN2395, NS(CpG‐ODN2395), or empty NS following incubation for 24 h. H) M2e‐specific IgG antibodies in BALB/C mice immunized with NS(M2e+cdGMP), NS(M2e+CpG), NS(M2e) + free cdGMP or NS(M2e) via the subcutaneous route. Error bars represent mean ± SEM (*N* = 5). I) M2e‐specific IgG antibodies in C57BL/6 mice or AGB6 mice immunized with NS(M2e+cdGMP). Error bars represent mean ± SEM (*N* = 3). Statistical analyses were performed by one‐way ANOVA or Student's *t*‐test (*p < 0.05, **p < 0.01, ***p < 0.001, ****p < 0.0001).

To assess the immunogenicity of the M2e nanoshell vaccine, lyophilized and reconstituted NS(M2e+cdGMP) was administered to mice in a single‐shot vaccination regimen. For mice inoculation, each Balb/C mouse was injected subcutaneously with 375 µg of nanoshells containing 10 µg of M2e peptides and 1.25 µg of cdGMP in 100 µL of PBS solution via the tail base. For comparison, free M2e peptides and M2e peptides mixed in 100 µL of commercial Alum salt adjuvants (≈400 µg of aluminum hydroxide) were administered in parallel. 42 days following the primary vaccination, sera were obtained from the immunized mice for peptide‐specific enzyme‐linked immunosorbent assay (ELISA) analysis (**Figure** **3**A). As compared to the control groups, single‐dose NS(M2e+cdGMP)s induced significantly higher titers of M2e peptide‐specific antibodies. Evaluation of the relative proportion of IgG1 and IgG2a revealed that the M2e nanoshell exhibited a balanced Th1 and Th2 response with a high level of IgG2a antibodies, whereas no IgG2a titer was observed in the control groups (Figure 3B and Figure S4, Supporting Information). As ADCC is the primary protective mechanism of anti‐M2e, which mediates secretion of the lytic enzyme from effector cells upon bridging with influenza‐infected cells,^[^ ^13^, ^16^ ^]^ we next assessed the binding capacity of vaccine‐induced anti‐M2e antibodies against Madin–Darby canine kidney (MDCK) cells infected two different influenza A viruses including A/Puerto Rico/8/1934 (PR8; H1N1) and A/HKx31 (H3N2). Immunostaining using sera derived from M2e nanoshell‐ or M2e/Alum‐immunized mice showed that whereas the serum from the M2e/Alum group showed negligible antibody binding to the infected cells, strong and broadly reactive antibody binding was observed from the serum of the M2e nanoshell group (Figure 3C). The ability of the antibody binding to elicit ADCC was assessed via a reporter cell‐based bioluminescence assay, and robust activation of luciferase reporter gene upon reporter cell co‐incubation with H1N1‐infected MDCK cells in the presence of M2e nanoshell serum confirmed antibody‐mediated cell bridging (Figure 3D).

In light of the robust anti‐M2e induction by the NS(M2e+cdGMP), we questioned if particulate adjuvancy alone in the absence of co‐encapsulated molecular adjuvant may be sufficient in improving M2e immunogenicity. To assess the contribution of the co‐encapsulated STING agonist, M2e nanoshells prepared with no adjuvant (NS(M2e)) or with an equivalent dose of co‐encapsulated CpG‐ODN ((NS(M2e+CpG‐ODN)) were prepared for comparison (Figure 3E). The immunogenicity of the nanoshell‐encapsulated adjuvants was first assessed using secreted embryonic alkaline phosphatase (SEAP) reporter cells that overexpress either the R232 isoform of human STING or the human TLR9 gene and the NS(cdGMP) and NS(CpG) showed superior and comparable immune stimulation to their respective free adjuvant counterparts (Figure 3F,G). While NS(M2e) and NS(M2e+CpG‐ODN) showed comparable cargo encapsulation and physicochemical properties as NS(M2e+cdGMP) (Figure 3E and Table S1, Supporting Information), these alternative formulations yielded significantly reduced anti‐M2e titers than the STING agonist‐loaded counterpart (Figure 3H). Of note, NS(M2e) adjuvated with free cdGMP of equivalent dosing to NS(M2e+cdGMP) showed no observable improvement in immunogenicity, which can be attributed to the low delivery efficiency of the free cyclic dinucleotides. Further assessment of NS(M2e+cdGMP) vaccination in C57BL/6 mice and AGB6 mice, a mouse strain with C57BL/6 background but deficient in IFN*α*/*β* and IFN*γ* receptors,^[^ ^17^ ^]^ showed that the M2e nanoshells' immunogenicity required proper interferon signaling (Figure 3I). These results highlighted the co‐incorporation of STING agonists in M2e nanoshells as an integral component for elevating M2e peptide immunogenicity.

### 2.3 M2e Nanoshell Vaccination Stimulates Th1 Helper T Cells, Follicular Helper T Cells, and Germinal Center Formation

**Figure 4:**
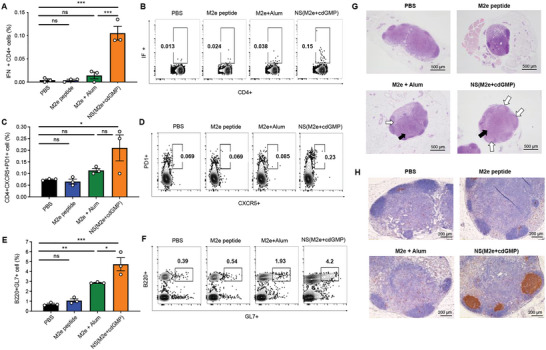
M2e STING agonist nanoshell promotes a lymph node environment favorable to Th1‐skewed antibody production. A,B) M2e‐specific IFN*γ* ^+^CD4^+^ T cell responses in immunized mice as determined by intracellular cytokine staining on day 7 following primary immunization. Error bars represent mean ± SEM (*N* = 3). C,D) Frequencies of and E,F) GL7+ germinal center B cells in the draining lymph nodes of immunized mice 14 days after immunization. Error bars represent mean ± SEM (*N* = 3). Statistical analyses were performed by one‐way ANOVA (*p < 0.05, **p < 0.01, ***p < 0.001). G) Perfused popliteal lymph nodes of immunized mice 14 days post‐vaccination were fixed and embedded in paraffin. Haematoxylin/eosin staining (H&E) was performed to identify follicular hyperplasia (white arrows) and paracortex hyperplasia (black arrows) in the lymph nodes. Scale bars = 500 µm. H) Popliteal lymph node sections were stained with anti‐GL‐7 antibodies (brown) for germinal center identification. Scale bars = 200 µm.

To gain mechanistic insight into the humoral responses to M2e, we assessed the induction of Th1 helper T cells, follicular helper T cells (*T*_FH_), and germinal center formation induced by vaccination with M2e peptides, Alum‐adjuvanted M2e peptides, and NS(M2e+cdGMP). Effector cell‐mediated ADCC is mainly triggered through engagement with Fc‐receptor (FcgR)IV (related to human FcgR)IIIa), which recognizes IgG2a in Balb/C mice. As IgG2a production is aided by Th1‐dependent IFN*γ* secretion,^[^ ^12^ ^]^ we first investigated M2e‐specific CD4^+^ T cell responses elicited by the different vaccine formulations. T cell responses were evaluated 7 days after vaccination by stimulating harvested splenocytes with M2e peptides. Following intracellular cytokine staining and flow cytometric analysis, the NS(M2e+cdGMP)‐vaccinated group showed the highest frequency of IFN*γ* ^+^ subset (**Figure** **4**A,B). In contrast, no significant difference in CD4^+^IFN*γ* ^+^ T cells was observed among the M2e+Alum and the control groups. The negligible CD4^+^IFN*γ* ^+^ T cell enhancement by Alum adjuvantation is consistent with the lack of IgG2a in the serum titer of the M2e+Alum group, and it highlights the role of Th1‐biased adjuvants for inducing ADCC‐favoring humoral responses. We next examined the presence of CD4^+^CXCR5^+^PD1^+^ *T*_FH_ in the draining lymph node (dLN) 14 days following vaccination. While M2e peptide vaccination in the presence and absence of Alum resulted in comparable levels of *T*_FH_ as the PBS control group, NS(M2e+cdGMP) inoculation significantly increased the *T*_FH_ population (Figure 4C,D). Likewise, analysis of the B lymphocyte population with GL7 activation markers, which are germinal center B cells that undergo rapid proliferation for antibody development and production, showed that M2e nanoshell vaccination induced the highest level of B220^+^GL7^+^ B lymphocytes (Figure 4E,F). Further examination of germinal center formation was performed via histological analysis of dissected lymph nodes 14 days following vaccination. In the lymph nodes of both M2e+Alum‐ and M2e nanoshell‐vaccinated groups, paracortical hyperplasia could be observed, which is characterized by infiltration of dendritic cells in the paracortex (Figure 4G). The lymph nodes of the M2e nanoshell‐vaccinated group also displayed prominent follicular hyperplasia, which is indicative of B cell proliferation and progressive development of germinal centers. Examination by immunohistochemistry further contrasts the GL‐7^+^ B cell distribution among the different vaccinated groups (Figure 4H). Prominent clustering of GL‐7+ B cells in the germinal center from the M2e nanoshell group indicates rapid B cell activation that favors subsequent plasma cell development. These results delineate the favorable lymph node environment and enhanced T cell helper functions induced by the STING agonist nanoshell for promoting anti‐M2e humoral responses.

### 2.4 Single‐Dose M2e Nanoshell Vaccination Confers Potent and Durable Protection against Lethal H1N1 Challenge

**Figure 5:**
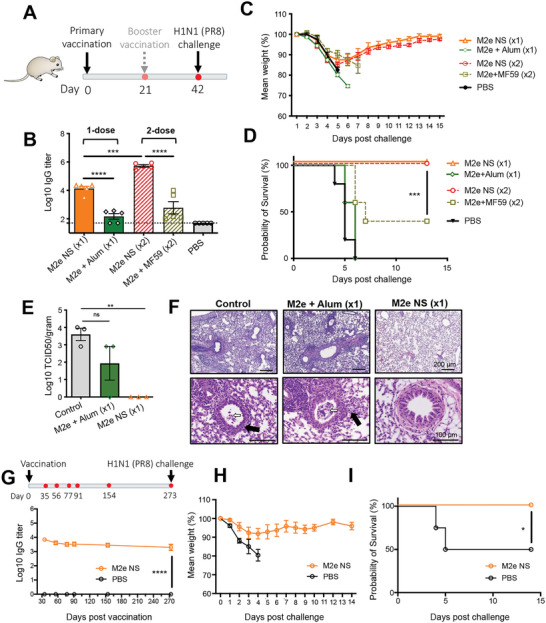
Assessment of antiviral protectivity by single‐shot M2e nanoshell vaccination. A) Vaccination schedule and viral challenge for single‐shot and prime‐boost immunization regimens. B) Assessment of anti‐M2e titers from mice serum collected on day 35 following the primary vaccination. C) Mouse body weight changes and D) survival rate after A/Puerto Rico/8/1934 (H1N1) infection upon challenge on day 42 with 3 × 10^5^ PFU viral dose via the intranasal route. (*N* = 5). E) Lung viral titers were evaluated 3 days following virus infection. (*N* = 3). F) Haematoxylin/eosin staining (H&E) was performed to identify lymphocytic cell infiltrates and perivascular inflammation (top panel). Scale bars = 200 µm. The lung tissues were also monitored for bronchiole injuries (bottom panel), including the presence of necrotic epithelial cells (white arrows) and airway wall thickening (black arrows). Scale bars = 100 µm. G) Anti‐M2e titers in mice following a primary M2e nanoshell vaccination over a 273‐day period. (*N* = 5). H) Mice were challenged on day 273 intranasally with 3 × 10^5^ PFU of A/Puerto Rico/8/1934 (H1N1) and assessed for (H) body weight changes and I) survival rate after infection. (*N* = 5). Error bars represent mean ± SEM. Statistical analyses were performed by one‐way ANOVA. The survival rate was analyzed by using the Log‐rank test (**p < 0.01, ***p < 0.001, ****p < 0.0001; ns, non‐significant).

To assess the protectivity conferred by the M2e nanoshell vaccine, we subjected immunized mice to a lethal PR8 influenza challenge. In addition to the single‐dose M2e nanoshell and the Alum‐adjuvanted M2e vaccine groups, we assessed two additional prime‐boost vaccine regimens with M2e nanoshells and M2e peptides adjuvanted with MF59, which is an oil‐in‐water emulsion adjuvant licensed for use in pandemic and seasonal influenza vaccines (**Figure** **5**A). M2e titer assessment showed that a booster M2e nanoshell vaccination increased M2e antibody levels by ≈2 orders of magnitude (Figure 5B). In contrast, a prime‐boost vaccination with MF59‐adjuvanted M2e peptides yielded lower anti‐M2e antibody titers than the single‐dose M2e nanoshell vaccination. Under the PR8 challenge, no observable protectivity was conferred by the one‐dose Alum‐adjuvanted M2e vaccination, and the 2‐dose MF59‐adjuvanted M2e vaccine conferred partial protection with 60% of the immunized mice dying from the viral challenge. In stark contrast, single‐dose M2e nanoshells fully protected the vaccinated mice (Figure 5C,D). Notably, although the 2‐dose nanoshell vaccine increased the overall humoral responses and serum antibody binding to influenza virus‐infected MDCK cells (Figure S5A, Supporting Information), equivalent ADCC activity, anti‐viral protectivity, and weight recovery profiles were observed between the prime‐boost and the single‐dose nanoshell regimens (Figure 5C,D and Figure S5B, Supporting Information), indicating that a plateau of anti‐M2e‐mediated protection was attained without the booster vaccination.

With the 1‐dose M2e nanoshell vaccination conferring comparable protection to the booster regimen, we further examined the extent of protectivity afforded by the single‐shot nanoshell vaccination. We collected the lung tissue from the PBS control, single‐shot M2e+Alum, and single‐shot M2e nanoshell groups 3 days following the influenza challenge. Viral load evaluation by 50% of tissue culture infective dose (TCID50) assay showed no detectable viral titer in the lungs of the M2e nanoshell group and indicated complete suppression of replicating viruses, whereas the control and M2e+Alum groups exhibited high pulmonary viral titers (Figure 5E). Histopathology analysis of the lung tissues further supported the nanoshells' prominent antiviral protectivity. Mice receiving the single‐dose nanoshell vaccine showed no observable pulmonary lesions. In stark contrast, the influenza challenge inflicted apparent pulmonary damages to the PBS control and the M2e/Alum groups, which showed severe tissue pathology with significant lymphocytic cell infiltrates and perivascular inflammation (Figure 5F). In addition, the bronchioles of the control groups showed necrotic epithelial cells and wall thickening, which are associated with the impairment of respiratory functions. The bronchioles of the M2e nanoshell‐vaccinated group were free of these pathological signs and showed normal histology with a thin‐walled airway and a columnar epithelium. These results demonstrate the M2e nanovaccine's exceptional protective effect in viral suppression and alleviation of virus‐induced lung damage. Of note, the antibodies mounted by the M2e nanoshells showed no neutralizing capacity against influenza viruses (Figure S6, Supporting Information), which highlights ADCC could effectively protect against pulmonary infectious diseases.

As waning antibody titers have been deemed the primary translational barrier for M2e‐based vaccine formulations, we further examined the durability of the humoral responses and protective effect conferred by the single‐shot M2e nanoshells over a 40‐week period. Remarkably, the anti‐M2e IgG levels remained steady over the observation period (Figure 5G). The prolonged humoral response suggests the induction of long‐lived plasma cells, the development of which is highly dependent on germinal center formation and helper T cell functions.^[^ ^18^ ^]^ On day 273 following the nanoshell vaccination, we assessed the nanoshell vaccine's protectivity by challenging the aged mice with a lethal dose of PR8 virus, and a control group of the same age was similarly challenged for comparison. Unlike the lethal challenge with the young mice that showed 100% mortality, the aged mice under the same lethal dose showed a 50% mortality. This reduction in influenza susceptibility can be attributed to lowered inflammation and immunopathology exhibited by aged subjects.^[^ ^19^ ^]^ Despite the reduced mortality, weight loss remained observable in the control group (Figure 5H). In comparison, M2e nanoshell‐vaccinated group had a 100% survival rate with a peak average weight loss of less than 10% (Figure 5H,I), demonstrating long‐lasting protectivity conferred by the single‐dose M2e nanoshell inoculation. The longevity of the nanoshell‐induced antibody stands in contrast to prior M2e vaccination efforts,^[^ ^7^ ^]^ which may owe their declining humoral responses to the difficulty in engaging the diminutive peptide antigens with cognate B cells in the lymph node follicles.

### 2.5 Nanoshell Enables Prolonged M2e Peptide Retention and Exposure in the Lymph Node Follicles for Antibody Induction

**Figure 6:**
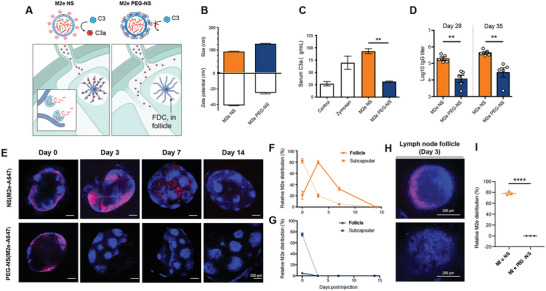
Spatiotemporal control of M2e peptide antigen distribution in the lymph node follicle by nanoshell carriers. A) Schematics illustrating the effect of nanoshell surface property on complement activation and lymph node distribution. B) Dynamic light scattering characterization of size and zeta potential of PEG‐free M2e STING agonist nanoshells (M2e NS) and PEG‐coated M2e STING agonist nanoshells (M2e PEG‐NS). (*N* = 3). C) The activated complement protein C3a concentration in BALB/c mouse serum (Control) and following incubation with zymosan (Zymosan), PEG‐free nanoshells (M2e NS), or PEGylated nanoshells (M2e PEG‐NS) (*N* = 3). D) M2e‐specific IgG titers in mice 35 days following immunization with M2e NS or M2e PEG‐NS. Error bars represent mean ± SEM (*N* = 5). Statistical analyses were performed by unpaired *t*‐tests. (**p < 0.01). E) BALB/c mice were inoculated with nanoshells containing fluorescent A647‐conjugated M2e peptide (M2e‐A647) for tracking of M2e antigen distribution over a 14‐day period. FDCs were labeled in situ with anti‐CD35 antibody and excised dLNs were cleared and imaged by confocal microscopy (CD35 blue; M2e‐A647 red). Scale bars = 200 µm. F,G) Co‐localization of M2e with subcapsular macrophages and lymph node follicles was evaluated via image analysis of M2e‐A647 signal coordination with lymph node boundaries and anti‐CD35 signals, respectively. H) Zoomed‐in visualization of M2e distribution in the lymph node follicles three days following subcutaneous administrated with either M2e NS or M2e PEG‐NS. Scale bars = 200 µm. I) Quantification of M2e NS and M2e PEG‐NS in the lymph node follicles 3 days following inoculation. (*N* = 3) (****p < 0.0001).

The M2e nanoshells' strong humoral responses and protectivity prompted us to question how the shielded peptides inside the nanocarrier could be displayed for B cell binding and antibody stimulation. As conventional nanocarrier‐based strategies rely on surface antigen display to enhance antigen engagement with cognate B cells,^[^ ^20^ ^]^ the counterintuitive nanoshell design and its performance evoked curiosity. We hypothesized that the nanoshells were capable of releasing M2e peptides in a prolonged fashion in the lymph node follicles for sustained immune stimulation (**Figure** **6**A). Induction of humoral immunity is facilitated by a network of FDCs in the lymph node follicles that present antigens to B cells for affinity maturation. As immune complexes formed by activated complement products and nanoparticulates can be relayed to the complement receptor‐rich FDCs following their capture by subcapsular macrophages,^[^ ^21^ ^]^ we envisioned that the surface of the surfactant‐free, anion‐rich nanoshells played a critical role in complement‐dependent FDC targeting. To test this hypothesis, we prepared a PEG‐coated nanoshell and compared the complement activation, follicle targeting, and immunogenicity between the PEG‐coated and PEG‐free nanoshells. PEG incorporation into the nanoshells was readily achieved via the addition of DSPE‐PEG in the oil phase during the nanoshell preparation. Compared to the PEG‐free nanoshells, PEG‐modified nanoshells (M2e PEG‐NS) contained equivalent M2e peptide and cdGMP encapsulation, possessed a slightly larger particle diameter (121 nm), but had a less anionic surface zeta‐potential at −24.9 mV (Figure 6B and Table S2, Supporting Information). To compare the complement activation between the M2e NS and the M2e PEG‐NS, we measured the level of anaphylatoxin C3a, a proteolytic product of the central complement protein C3,^[^ ^22^ ^]^ following particle incubation in mice serum. The M2e NS induced significant complement activation, resulting in a C3a level comparable to that of the zymosan positive control. In contrast, PEG modification completely suppressed the complement activation with PEG‐NS yielding similar C3a levels as the control serum (Figure 6C). Anti‐M2e titer assessment 28 and 35 days following immunization of the two M2e nanoshells in mice showed a direct correlation between the nanoshells' complement activation and their vaccine performance, with PEG coating reducing the overall anti‐M2e titers by more than an order of magnitude (Figure 6D). These results show that the M2e immunogenicity can be significantly altered by the surface property of the antigen carrier.

To examine the M2e distribution upon encapsulation and delivery by the two different nanoshells, we performed whole‐tissue fluorescence measurement of fluorescently labeled M2e peptides in the dLNs. Alexa Fluor 647 dye‐conjugated M2e peptides encapsulated in either PEG‐free nanoshells (NS(M2e‐A647)) or PEG‐coated nanoshells (PEG‐NS(M2e‐A647)) were delivered into mice via footpad injection, and the popliteal lymph nodes excised at different time points were treated using an X‐CLARITY Tissue Clearing System to obtain optically transparent lymph nodes prior to fluorescence examination^[^ ^23^ ^]^ (Figure S7, Supporting Information). Examination 4 h post nanoshell administration showed that both NS(M2e‐A647) and PEG‐NS(M2e‐A647) resulted in M2e localization at the boundary area of the lymph nodes, which is indicative of nanoparticle capture by the subcapsular sinus macrophages^[^ ^24^ ^]^ (Figure 6E). In contrast, no detectable fluorescence signal was observed in the lymph node following the administration of free M2e‐A647 peptides (Figure S8, Supporting Information). Despite efficient lymph node targeting by both M2e NS and M2e PEG‐NS, observable differences emerged upon examination of antigen retention 3 days following nanoshell administration. NS(M2e‐A647) administration resulted in a high level of M2e antigen co‐localization at the lymph node follicles, whereas PEG‐NS(M2e‐A647) were largely cleared with no detectable antigen signals in the lymph node. Further examination of M2e distribution from the NS(M2e‐647) group showed an intriguing shift in the distribution pattern on day 7. With a decline in follicle‐bound antigen signals, venule‐like fluorescence patterns emerged. The venule‐like patterns are reminiscent of the mesh‐like structure of the lymph node conduit,^[^ ^25^ ^]^ which is an interconnected network that allows for the passage of low molecular weight molecules (<70 kDa) between afferent lymphatic vessels, follicles, and high endothelial venules. As the 100‐nm nanoparticulates are too large to access these channels, antigen distribution in these conduit channels reflects that the small peptide antigens had been released from the nanocarriers and were being exported from the follicles via the conduit system. The timing of this pattern emergence is consistent with the release kinetics of the nanoshells, which possess a sustained antigen release profile over several days (Figure 6F). By day 14, a trace amount of antigen signal remained detectable in the lymph node follicle for the NS(M2e‐A647) group. The contrasting kinetics in lymph node retention between the PEG‐coated and non‐modified nanoshells provide mechanistic insights into the discrepancies between the two nanoparticles' immunogenicity (Figure 6F,G). To further assess the distribution of the nanoshells in the lymph node follicles, we closely examined the follicles 3 days post‐nanoshell administration. NS(M2e‐A647) showed a polarized distribution in the follicle (Figure 6H), manifesting a localization pattern consistent with the distribution of FDCs in germinal centers upon immune activation.^[^ ^26^ ^]^ On the other hand, no antigen retention in the follicle was observed in the PEG‐NS(M2e‐A647) group (Figure 6H,I). We further showed that injection with a cobra venom factor (CVF), a snake toxin that depletes complement factors, significantly impaired the nanoshells' follicle‐targeting ability in mice (Figure S9, Supporting Information). Altogether, these results demonstrate that nanoshell‐encapsulated M2e antigens can be retained and released in the FDC network for prolonged B cell stimulation in a complement‐dependent manner, highlighting unique nanoshell surface and antigen release attributes that contributed to the nanovaccine's single‐dose efficacy.

### 2.6 Single‐Dose M2e Nanoshell Vaccine Confers Broad Protectivity against Heterosubtypic Influenza Viruses

**Figure 7:**
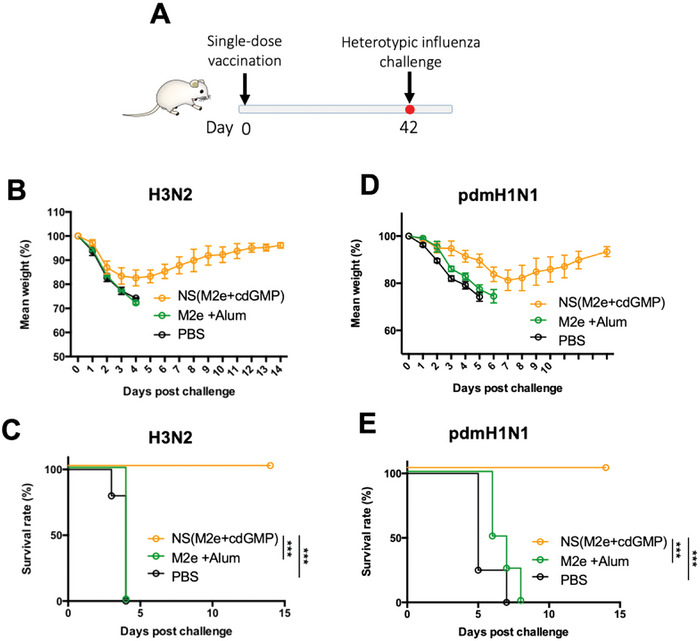
Assessment of M2e nanoshell vaccine against heterotypic influenza challenge. A) Vaccination and viral challenge schedule. Mice were subcutaneously inoculated with PBS, M2e NS, or free M2e peptides adjuvanted with Alum. The mice were challenged on day 42 intranasally with 3 × 10^6^ PFU influenza A/HKx31 (H3N2) or 2.5 × 10^6^ PFU pandemic 2009 H1N1 (pdmH1N1). B) Mouse body weight changes and C) survival rate after influenza A/HKx31 (H3N2) infection. D) Mouse body weight changes and E) survival rate after the influenza pandemic 2009 H1N1 (pdmH1N1) infection. Error bars represent mean ± SEM. (*N* = 5). The survival rate was analyzed by using the Log‐rank test (*** *p* < 0.001).

With the collective molecular and particulate adjuvancy of the M2e STING agonist nanoshells contributing to the nanovaccine's robust and long‐lasting anti‐M2e titers, we then examined the protective efficacy against heterosubtypic influenza viruses by the single‐dose nanoshell regimen (**Figure** **7**A). We first examined the vaccine's protectivity against the HKx31 strain, which is an H3N2 variant virus that has a conserved M2e sequence to the previously examined H1N1 virus. Under the viral challenge, the M2e nanoshell vaccination fully protected the mice from mortality, whereas the Alum‐adjuvanted vaccine control conferred no observable protectivity or survival benefit with all mice dying within 4 days post‐challenge (Figure 7B,C). Further antiviral assessment was performed with the pandemic 2009 H1N1 strain (pdmH1N1), which notably has as many as 4 M2e amino acid residues that differ from the 23‐peptide long M2e antigen used for the nanoshell vaccine (Table S3, Supporting Information). Despite the differences in the peptide sequences, the single‐dose nanoshell vaccine remained fully protective against pdmH1N1 (Figure 7D,E), highlighting the broad applicability of the nanoshell vaccine against heterosubtypic influenza viruses.

## 3 Discussion

To overcome the low immunogenicity of M2e antigen for influenza vaccination, various antigen modification strategies have brought forth M2e‐based fusion proteins with carrier proteins,^[^ ^27^ ^]^ strong immunogens,^[^ ^8^, ^28^ ^]^ and immune cell targeting ligands.^[^ ^10^ ^]^ To our knowledge, a single‐dose vaccine formulation conferring full protection against heterotypic influenza challenges has not been previously achieved. We demonstrate the rational integration of molecular and particulate adjuvancy in vaccine designs for enhancing the ADCC activity of M2e antigens using asymmetrically stabilized polymeric nanoshells. High‐density M2e antigen and STING agonist co‐encapsulation in anionic nanoshells enabled broad and durable anti‐influenza protectivity under a single‐dose vaccine regimen, which has immense public health implications and values. In contrast to the predominant strategy of fusion protein design for enhancing M2e immunogenicity, the adoption of 23‐amino‐acid long M2e peptides in the present work offers scalability advantages as the peptides can be readily synthesized via solid‐phase peptide synthesis. Importantly, we showed that the peak of anti‐M2e antibody‐mediated protectivity can be attained following a single‐dose nanoshell inoculation. A booster shot of the nanoshell vaccine did not confer apparent protective benefits despite raising the anti‐M2e titers by two orders of magnitude. The plateauing protectivity can be explained by the action mechanism of anti‐M2e, which serves as a bridge between infected cells and effector cells for the stimulation of ADCC. Unlike neutralizing antibodies that rely on pathogen binding for virus neutralization, ADCC‐inducing antibodies intercept virus replication by lysing infected cells via stimulation of perforins and granzymes from effector cells. The activity of ADCC‐inducing antibodies typically forms a sigmoidal relationship with the lytic function of effector cells, which exhibit saturating, maximal cytotoxicity upon reaching a specific antibody concentration.^[^ ^29^ ^]^ Such concentration is inversely correlated with antibody affinity and reflects the antibody coating density on target cells required to fully activate effector cells. The observed plateau of anti‐M2e protectivity in mice indicates that the titers achieved by the single‐dose regimen provided sufficient coverage of the infected cells under the lethal challenge, enabling effective effector cell recruitment for viral clearance. Achieving such a level with peptide antigens under a single dose regimen attests to the extraordinary adjuvancy effect of the follicle‐targeting STING agonist nanoshell. Such robust ADCC‐inducing capability may have therapeutic implications for other respiratory pathogens and cancers.

Our study also highlights the potency of STING agonist adjuvant in boosting Th1‐skewed humoral responses for ADCC induction. Activation of STING by cyclic dinucleotides directly phosphorylates IRF3 and in turn stimulates the expression of type I interferons,^[^ ^30^ ^]^ which has a profound impact in shaping the adaptive immune responses. Although the STING agonist adjuvant has drawn great interest in vaccine development against infectious pathogens and cancers,^[^ ^31^ ^]^ the utility of cyclic dinucleotides and assessment of their adjuvancy effect to alternative adjuvants have been challenging owing to the poor intracellular delivery efficiency of the compound. Similar to other nanocarriers that have been designed to enhance STING agonist delivery,^[^ ^20^, ^32^ ^]^ the polymeric nanoshells in the present work are capable of enhancing lymph node targeting and immune cell uptake of the hydrophilic molecules.^[^ ^11^, ^15^ ^]^ We herein further demonstrate a nanoshell‐based comparison between cdGMP and CpG‐ODN 1826, an alternative adjuvant that exerts its adjuvant function via the activation of TLR9. Upon unifying the dosing and delivery profiles of cdGMP and CpG‐ODN for M2e nanoshell preparations, cdGMP proved to significantly outperform CpG‐ODN in enhancing anti‐M2e titer production. The reduced humoral responses by the class B CpG‐ODN may be attributed to its lower capacity in stimulating type I IFN and its tendency to induce low‐affinity short‐lived plasma cells.^[^ ^33^ ^]^ Our observation is consistent with a recent M2e vaccine study, which shows that CpG‐ODN adjuvantation underperforms as compared to poly(I:C),^[^ ^10^ ^]^ which is a TLR3 agonist that activates IRF3 similarly as cdGMP for type I IFN induction.^[^ ^34^ ^]^ With type I IFN capable of enhancing humoral responses via multiple mechanisms, including the promotion of CD4^+^ T cell activation, stimulation of and enhancement of germinal center formation,^[^ ^35^ ^]^ we verified these type I IFN‐associated attributes in STING agonist nanoshell‐inoculated mice. The lymph nodes of the nanoshell‐inoculated mice displayed elevated populations of Th1, *T*_FH_, and GL7^+^ germinal center B cells. These cellular populations collectively favor the development and maturation of long‐lived plasma cells that are conducive to the establishment of durable humoral responses.

Another factor underscoring the prominent nanoshell immunogenicity is its capability to prolong the exposure of M2e peptide antigens in the lymph node follicles. Sustained antigen retention in the germinal center helps direct affinity maturation and survival of cognate B cells during the rapid proliferation of germinal center B cells,^[^ ^36^ ^]^ and efforts to enhance humoral responses have prompted emerging vaccination strategies based on slow‐delivery immunization strategies and designer delivery systems.^[^ ^37^ ^]^ We demonstrate that shielding peptide antigens in a biodegradable nanoshell casing rather than coating them onto nanoparticle surfaces conferred an unexpected spatio‐temporal control over antigen distribution in the lymph node follicles. The anionic polymers adopted for asymmetric emulsion stabilization and the surfactant‐free nature of the nanoshells bestowed the particles a highly anion‐rich surface, which can activate the complement system through the classical pathway in the presence of calcium ions.^[^ ^38^ ^]^ The anionic nanoshells can efficiently target FDCs in a complement‐dependent manner, and the inclusion of a commonly used PEG stabilizer abrogated the complement activity and follicle‐targeting ability. Upon delivery to the FDC network, nanoshell degradation enabled sustained peptide antigen exposure in the germinal center for B cell stimulation. The revelation of the antigen shuttling mechanism adds a new design principle to the vaccine paradigm that typically anchors antigens on particulate surfaces for B cell engagement. Compared to typical nanoparticulate vaccines whose surface‐bound antigens and moieties could influence their complement activation and follicle targeting capability,^[^ ^39^ ^]^ encapsulating antigens in a degradable anionic nanocapsule presents a versatile alternative for directing antigens to the lymph node follicle. In light of the recent discovery that antigens can encounter extracellular proteases that lead to epitope breakdown prior to reaching the lymph node follicles,^[^ ^40^ ^]^ nanoshell‐encapsulated antigens may offer the added advantage of antigen protection as compared to nanoparticulate vaccines surface‐displayed antigens. Further tuning of capsule degradability may offer broader control over antigen durability in the germinal center, which could add another dimension in vaccine design toward enhancing adaptive immunity.

## 4 Conclusion

In conclusion, our work demonstrates a highly effective M2e nanovaccine that achieves broad and durable influenza protection under a single dose regimen. The collective adjuvancy effect of STING agonist and nanoparticle‐mediated antigen retention in the FDC led to a profound elevation in antigen immunogenicity. The nanoshell vaccine makes possible the simplification of M2e antigen design, enabling the preparation of a translationally viable vaccine formulation based on 23‐amino‐acid long peptide antigens. The present study further offers mechanistic insights and design inspirations for the delivery of peptide antigens, adding to the arsenal of nanotechnology toolsets for pandemic preparedness.

## 5 Experimental Section

### Ethical Statement

All animal experiments were performed under an approved Institutional Animal Care and Use Committee (IACUC) protocol (# 15‐12‐893) in Academia Sinica, Taiwan.

### Cells and Viruses

MDCK was purchased from the Bioresource Collection and Research Center (Hsinchu, Taiwan). MDCK cells were maintained in Dulbecco's Modified Eagle medium (DMEM) (Invitrogen) with 10% of fetal bovine serum (FBS) (Invitrogen, Carlsbad, CA) and 1% of penicillin/streptomycin/amphotericin B (PSA) (Invitrogen), and cultured in 37 °C and 5% CO2. When influenza virus infection in MDCK cells, the infection medium (DMEM containing 0.075% BSA, 1% non‐essential amino acid, 1% sodium pyruvate, 1% HEPES, 1% PSA, and 2 µg mL^−1^ TPCK‐treated trypsin) was used. Influenza A virus strain A/Puerto Rico/8/1934 (H1N1) was kindly provided by Professor Shin‐Ru Shih at Chang Gung University. Influenza A virus strain A/HKx31 (H3N2) was kindly provided by Professor Hung‐Chih Yang at the National Taiwan University College of Medicine, and A/California/7/2009 (pdmH1N1) was kindly provided by Professor Li‐Min Huang at National Taiwan University Hospital. All viruses were propagated in the allantoic cavity of 10‐day‐old specific pathogen‐free (SPF) chicken embryos (JD‐SPF Biotech, Miaoli, Taiwan). Virus titer was determined by plaque assays as previously described.^[^ ^41^ ^]^

### M2e Nanoshell Preparation

For the present study, a consensus M2e peptide sequence [SLLTEVETPIRNEWGCRCNDSSD] was adopted (Genescript; purity >95%). The nanoshells were prepared by an optimized water‐oil‐water double emulsion following previously reported protocols.^[^ ^11^, ^15^ ^]^ The inner aqueous phases were prepared by dissolving desired encapsulants in 200 mm NaHCO_3_ buffer. Polymer solutions were prepared by dissolving 75 mg mL^−1^ of carboxyl‐terminated, 50:50 PLGA (Mw 7000–17 000; Sigma‐Aldrich) in ethyl acetate. For a typical preparation of M2e nanoshell vaccine, 20 µL of an aqueous solution containing 40 mg mL^−1^ of M2e peptides and 5 mg mL^−1^ of cdGMP (InvivoGen) was emulsified in 200 µL of polymer solution in ice with an Ultrasonic Probe Sonicator in the pulse mode with 40% amplitude and on–off durations of 1 and 2 s for 1 min. The first emulsion was subsequently added to 5 mL of 10 mm NaHCO_3_, which was then probe sonicated at 30% amplitude with on–off durations of 1 and 2 s for 2 min. The emulsion was subsequently poured into 8 mL of water and heated at 40 °C under gentle stirring in a fume hood for solvent evaporation. After 1 h of solvent evaporation, the nanoparticles were collected using 100 kDa molecular weight cut‐off (MWCO) Amicon filters (Sigma‐Aldrich) to remove unencapsulated materials. For non‐asymmetrically stabilized nanoemulsions, ester‐terminated PLGA (lactide:glycolide 50:50, Mw 7000–17 000; Sigma‐Aldrich) was used as the non‐charged polymer. The condition in which the absence of differential ionic buffers was generated by replacing the inner aqueous buffer of 10 mm NaHCO_3_. For the NS(M2e+CpG‐ODN) and NS(M2e), the inner aqueous phase was replaced with 20 µL of 40 mg mL^−1^ M2e peptide with 5 mg mL^−1^ of CpG‐ODN 1826 (InvivoGen) and 40 mg mL^−1^ M2e peptide, respectively. For PEG‐coated M2e nanoshell preparation, the oil phase was replaced with 200 µL of ethyl acetate containing 50 mg mL^−1^ of carboxyl‐terminated PLGA and 10 mg mL^−1^ of 1,2‐Distearoyl‐sn‐Glycero‐3‐phosphoethanolamine conjugated PEG (DSPE‐PEG(2000)‐OH; Nanocs). For nanoshells encapsulating fluorescently labeled M2e peptide antigens, M2e peptides were synthesized with Alexa Fluor 647 conjugated to its N‐terminus (Creative peptides). The collected nanoparticles were evaluated with dynamic light scattering, nanoparticle tracking analysis, cryoEM, and Micro BCA assay (Micro BCA Protein Assay Kit; ThermoFisher Scientific), and HPLC for physicochemical properties, particle concentration, particle morphology, peptide encapsulation efficiency, and cdGMP encapsulation, respectively. Lyophilized M2e nanoshell vaccines were prepared by suspending the nanoshells in 10 mm disodium phosphate and 25% sucrose at a concentration of 50 mg mL^−1^ prior to freezing and lyophilization. Prior to each immunization study, the nanoparticles were reconstituted and diluted to desired concentrations with water and osmotically adjusted with sucrose solution.

### Antigen and Adjuvant Release Kinetic Studies

The antigen and adjuvant release kinetics in physiologically relevant conditions were characterized with a dialysis experiment in two different pH environments (pH 5 and 7), in which M2e nanoshells were loaded in dialysis tubes (10 kDa MWCO, Slide‐A‐Lyzer MINI Dialysis Device, Thermo Fisher Scientific) and collected at predetermined time points for M2e peptide and cdGMP quantification.

### Animal Immunization and Serum Collection

With the exception of the comparison between C57BL/6 and AGB6 mice, all animal studies were performed with BALB/c mice. SPF BALB/c 7‐week‐old female mice and C57BL/6 mice were purchased from National Laboratory Animal Center, Taipei, Taiwan. AGB6 mice were kindly provided by Dr. Lin, Yi‐Ling at the Institute of Biomedical Sciences, Academia Sinica, Taiwan. Mice were housed in the animal facility maintained by the Institute of Biomedical Sciences, Academia Sinica. For nanoshell vaccination, mice were subcutaneously (s.c.) immunized via the tail base with 10 µg/dose of M2e peptides and 1.25 µg/dose of cdGMP nanoparticles in 100 µL of the solution containing 250 µg of nanoshells. Free M2e peptides were administered with 10 µg of M2e peptides solubilized in 100 µL of PBS. For Alum‐adjuvant M2e peptides, 10 µg of M2e peptides were mixed in 100 µL of commercial Alum salt adjuvants (≈400 µg of aluminum hydroxide) for administration. For the MF59‐adjuvanted vaccine, 10 µg of M2e in 50 µL of PBS was mixed with 50 µL of MF59 (AddaVax; InvivoGen) for administration. Prime‐boost regimens were given with a 21‐day interval between the primary and booster vaccination. For serum collection, blood was collected from the facial vein into BD SST microtainers (BD Biosciences) at designated time points. Sera were obtained after centrifugation at 3000 × *g* for 10 min and stored at −20 °C.

### ELISA

Flat‐bottom microplates (Nunc, Denmark) were coated overnight with M2e peptide antigens (100 ng/well) at room temperature. After washes, 5% (w/v) skim milk (BD Difco, Sparks, MD) in PBST (with 0.05% Tween 80) was added to the wells for blocking for 1 h. After blocking, mouse sera derived at predetermined time points were added to the wells and incubated for 1 h at room temperature. After repeated washes, secondary antibodies, including goat anti‐mouse IgG HRP conjugate (Jackson ImmunoResearch), goat anti‐mouse IgG1 HRP conjugate (Abcam), or goat anti‐mouse IgG2a HRP conjugate (Abcam), was added and incubated for 1 h. After further washes, 100 µL of 3,3′,5,5′ tetramethylbenzidine (TMB) microwell Peroxidase Substrate (KPL, Gaithersburg, MD) was dispensed to each well and incubated for 10 min in the dark. Finally, 100 µL of TMB stop solution (KPL, Gaithersburg, MD) was used to stop the reaction. The optical density at 450 nm was read using a spectrophotometer (ThermoFisher Scientific). M2e‐specific titers were calculated based on end‐point titers.

### Immunofluorescence Antibody Assay

MDCK cells were seeded into 96‐well tissue culture plates at a density of 1.2 × 10^4^ cells per well. After incubation for 24 h, the MDCK cells were washed twice with infectious medium and then infected with MOI = 1 of H1N1 or H3N2 virus. Following incubation for 24 h, the infected MDCK cells were washed twice with PBST, and then fixed by the addition of cold 80% acetone. Following 20 min of incubation with acetone at −20 °C, acetone‐fixed, infected, and un‐infected MDCK cells were blocked with 100 mL of 1% BSA‐PBS for 2 h at room temperature. After blocking, 50 µL of pooled sera (1:200 dilution in 1% BSA‐PBST) was added to the wells and incubated for 1 h at room temperature. The wells were then washed with PBST three times, and 50 µL of FITC‐tagged anti‐mouse secondary antibody (1:400 dilution) was added into the wells and incubated for 1 h at room temperature. 50 µL of DAPI (1:400 dilution) was added directly into the wells and incubated with a secondary antibody for 15 min at room temperature. The plates were then washed with PBST three times (5 min) and covered with glycerol. Fluorescence was then visualized via fluorescence microscopy (Olympus IX‐83).

### ADCC Surrogate Assay

The ADCC reporter bioassay was performed according to the manufacturer's protocol (Cat No. G7015, Promega). Briefly, the MDCK cells were infected by PR8 H1N1 using an infection medium (DMEM containing 0.075% BSA, 1% non‐essential amino acid, 1% sodium pyruvate, 1% HEPES, 1% PSA, and 2 µg mL^−1^ TPCK‐treated trypsin) at 37 °C and 5% CO_2_. The H1N1‐infected MDCK cells were harvested and seeded in sterile white 96‐well plates (Corning) 24 h before the assay. Serum samples derived from mice were heat inactivated for 30 min at 56 °C and then fivefold serially diluted in assay buffer (RPMI 1640 containing 4% ultra‐low IgG FBS). The serum dilutions and a stable Jurkat cell line expressing mouse Fc*γ*R (Cat No. G7015, Promega) were added to the infected MDCK cell‐seeded wells and incubated for 6 h at 37 °C at an effector:target cell ratio of 5:1. Cells were equilibrated to room temperature for 15 min prior to the addition of Bio‐Glo Luciferase assay substrate (Promega). Luminescence was then quantified after 10 min of incubation using GloMax (Promega). Data were expressed as luminescence RLU of signal in the absence of serum.

### Reporter Cell Assay for STING and TLR9 Activation

The activity of human STING or TLR9 genes was quantified using 293‐Dual hSTING‐R232 cells (InvivoGen) and HEK‐Dual hTLR9 cells (Invivogen), respectively. The viability of the reporter cells was first validated using TOOLS Cell Counting (CCK‐8) Kit (BIOTOOLS Co., Ltd., Taipei, Taiwan). To perform the reporter assay, free cdGMP (6 µg), NS(cdGMP) (6 µg cdGMP, 0.75 mg PLGA), free CpG‐ODN2395 (2 µg), NS(CpG) (2 µg CpG, 0.25 mg PLGA), and empty NS (0.75 mg PLGA or 0.25 mg PLGA, equivalent with compared NS) were prepared in 20 µL of PBS. Serially diluted sample solutions were prepared in parallel. The sample solutions were added to 180 µL of culture media containing 1 × 10^5^ of either 293‐Dual hSTING‐R232 cells or HEK‐Dual hTLR9 cells in a 96‐well plate. The plate was incubated at 37 °C in a CO_2_ incubator for 24 h, and the supernatant was collected for evaluation of SEAP activity upon the addition of QUANTI‐Blue solution (InvivoGen). The absorbance at 650 nm was measured using a spectrophotometer (ThermoFisher Scientific).

### Intracellular Cytokine Staining and Flow Cytometric Analysis

Intracellular cytokine staining for identifying M2e‐specific CD4^+^IFN*γ* ^+^ helper T cells was performed using splenocytes derived 7 days following vaccination. Single cells were prepared from spleens and plated at 1 × 10^6^ cells/well into round‐bottom 96‐well plates. 2 µg of M2e peptide antigens was added at 37 °C with 5% CO_2_ to stimulate CD4^+^ T cells. After 4 h, GolgiPlug protein transport inhibitor (BD Biosciences, San Jose, CA) was added. Plates were incubated for another 4 h and then spun at 4 °C to remove the medium. Cells were re‐suspended in 40 µL of 1:400 diluted anti‐CD4‐PE‐Cy7 (clone RM4‐5; BD Biosciences) antibodies. After 30 min of incubation on ice, cells were washed, re‐suspended in 100 µL of Cytofix/Cytoperm solution, and incubated on ice for 20 min. After two washes, cells were stained with 40 µL of 1:200 diluted anti‐IFN*γ* APC antibody (clone XMG1.2; BD Biosciences) overnight at 4 °C. Cells were washed three times before acquisition using a FACS LSR II (Institute of Biomedical Sciences, Academia Sinica). Analysis was done by FlowJo software. Backgrounds as determined for samples without peptide stimulation were subtracted from the values presented for test samples.

Assessment of follicular helper T cells and germinal center B cells was performed 14 days after the primary vaccination. Inguinal lymph nodes were collected from euthanized mice followed by tissue digestion into single‐cell suspensions, and 1 × 10^6^ cells were transferred into each well of a round‐bottom 96‐well plate. The cells were first incubated with anti‐mouse CD16/CD32 (clone 2.4G2; BD Biosciences) for 30 min to block Fc receptors. Following Fc blocking, the cells were incubated with antibodies corresponding to specific surface markers. For follicular helper T cell assessment, the staining antibodies included anti‐CD3‐APC (clone 17A2; eBiosciences), anti‐CD4‐PE‐Cy7 (clone RM4‐5; BD Biosciences), anti‐PD1‐PerCP‐eF710 (clone J43; eBiosciences), and anti‐CXCR5‐PE‐CF594 (clone 2G8; BD Biosciences). For GL7+ germinal center B cell assessment, the staining antibodies included anti‐B220 (human/mouse) PE (clone RA3‐6B2; BioLegend), and anti‐GL7‐Pacific Blue (clone GL7; BioLegend). After two washes, the cells were resuspended in PBS containing 2% FBS followed by acquisition using a FACS LSR II (BD Biosciences). Analysis was done by the FlowJo software (Flowjo LLC, Ashland, OR).

### Tissue Histology and Immunohistology

Popliteal lymph nodes and lungs were dissected from mice and fixed with 10% formalin. For histological analysis, the samples were stained with hematoxylin and eosin. For examining GL7^+^ B cells in the lymph nodes, paraffin‐embedded tissues were sectioned and deparaffinized with xylene and rehydrated with ethanol in water. The samples were then incubated in a hot citrate buffer for 20 min and subsequently cooled to room temperature. The samples then underwent peroxidase quenching with 3% hydrogen peroxide and avidin/biotin blocking via a commercial blocking kit (Vector Laboratories). After blocking, the tissues were stained with antibodies against GL7 (2.5 µg mL^−1^, BioLegend Cat #144 601). The antibody‐coated tissue sample was then treated using a commercial immunohistochemistry kit (DAB 2‐component Kit; C09‐100; OriGene) following the manufacturer's protocol.

### Hemagglutination Inhibition Assays

Serum was mixed with PR8 H1N1 virus for 30 min, following which 1% chicken RBCs were added to the mixture and incubated for 45 min at room temperature. A hemagglutination inhibition (HAI) titer was defined as the highest serum dilution factor leading to HAI; samples without any detectable HAI activity were assigned a titer of <10.

### Influenza Viral Challenge

All viral challenges were performed via intranasal inoculation. Mice were first anesthetized by isoflurane anesthetics, and they were inoculated with 25 µL of viral solutions. For lethal viral challenges, PR8 was administered at a dose of 3 × 10^5^ PFU, A/HKx31 (H3N2) was administered at 3 × 10^6^ PFU, and A/California/7/2009 (pdmH1N1) at 2.5 × 10^6^ PFU.

### Viral Load Assessment

Lung tissues were collected from mice 3 days post‐viral challenge, and the tissues were placed into an infection medium for homogenization by sonication. After sonication, the supernatant was collected following centrifugation at 3000 × *g* for 30 min. Viral load was evaluated by 50% of tissue culture infective dose (TCID50) assay. Briefly, lung homogenate supernatants were first serially diluted with an infection medium. MDCK cells were seeded in 96‐well microplates (1 × 10^4^ cells/well) and cultured for 24 h at 37 °C. 50 µL of the supernatants were then added to the cells and incubated for 1 h at 37 °C. After the infection, the media was removed and the cells were washed with PBS. 100 µL of fresh infection media was then added to the cells and incubated for 4 days at 37 °C. The culture media was then harvested, which was then mixed with 1% chicken RBC at a 1:1 ratio for hemagglutination spot assay for viral titer assessment.

### Complement Activation Assay

Complement activation was assessed via the quantification of the C3a product. Mice serum was first incubated with designated samples (zymosan, M2e NS, or M2e PEG‐NS) for 2 h at 37 °C. For detection of murine C3a, flat‐bottom 96‐well plates (Nunc Denmark) were incubated with 5 µg mL^−1^ of the capture antibody rat anti‐mouse C3a (clone I87‐1162; BD Pharmagen) overnight at 23 °C. After blocking, 1:200 serum samples or mouse C3a protein (BD Pharmagen) were added to the wells and incubated for 1 h. C3a content was determined by sequential incubation with biotin rat anti‐mouse C3a (clone I87‐419; BD Pharmagen), 1 µg mL^−1^ streptavidin‐horseradish peroxidase (Pierce), OptiEIA TMB substrate (BD Pharmagen), and 2 m H_2_SO_4_.

### Immunogen Tracking in Lymph Node Follicles

For examining M2e antigen distribution in the lymph node, mice were inoculated with 50 µg of PEG‐free or PEG‐coated nanoshells containing 2 µg of Alexa‐Fluor 647‐tagged M2e peptides via footpad injection. 18 h prior to lymph node excision, mice were injected subcutaneously in the footpad with 4 µg BV421‐labeled anti‐CD35 (BD Biosciences 740 029) for in situ labeling of lymph node follicles. Popliteal lymph nodes were then processed via tissue clearing. The lymph nodes were fixed overnight at 4 °C in 4% paraformaldehyde and subsequently washed in 1× PBS for 24 h at 4 °C to remove formaldehyde residues. The sample was then submerged in a solution containing 25% (w/v) X‐CLARITY Polymerization Initiator and X‐CLARITY Hydrogel Solution (Logos Biosystems) at a 1:100 ratio at 4 °C for 24 h. Following polymerization with the X‐CLARITY Polymerization System, the hydrogel‐embedded tissue was placed into Electrophoretic Tissue Clearing Solution for passive tissue clearing. The resulting lymph node was imaged via confocal microscopy.

### Statistical Analyses

Data were analyzed by Student's *t*‐test or ANOVA followed by Dunnett's multiple comparison tests using GraphPad Prism. A *p*‐value smaller than 0.05 was considered significant.

## Conflict of Interest

The authors declare no conflict of interest.

## Supporting information

Supporting Information

## Data Availability

The data that support the findings of this study are available in the supplementary material of this article.
