# Supplementary material for: Lymph Node Follicle‐Targeting STING Agonist Nanoshells Enable Single‐Shot M2e Vaccination for Broad and Durable Influenza Protection
Source: Adv Sci (Weinh). 2023 Apr 24;10(17):2206521. doi: 10.1002/advs.202206521 (PMC10265066; doi:10.1002/advs.202206521)
Supplement: Supplementary file 1 — Supporting Information [file ADVS-10-2206521-s001.pdf]

ADVANCED SCIENCE
Open Access

Supporting Information

for *Adv. Sci.*, DOI 10.1002/advs.202206521

Lymph Node Follicle-Targeting STING Agonist Nanoshells Enable Single-Shot M2e Vaccination for Broad and Durable Influenza Protection

Hsiao-Han Tsai, Ping-Han Huang, Leon CW Lin, Bing-Yu Yao, Wan-Ting Liao, Chen-Hsueh Pai, Yu-Han Liu, Hui-Wen Chen and Che-Ming J. Hu**

Supporting Information

Lymph node follicle-targeting STING agonist nanoshells enable single-shot M2e vaccination for broad and durable influenza protection

Hsiao-Han Tsai, Ping-Han Huang, Leon CW Lin, Bing-Yu Yao, Wan-Ting Liao, Chen-Hsueh Pai, Yu-Han Liu, Hui-Wen Chen, Che-Ming J. Hu**

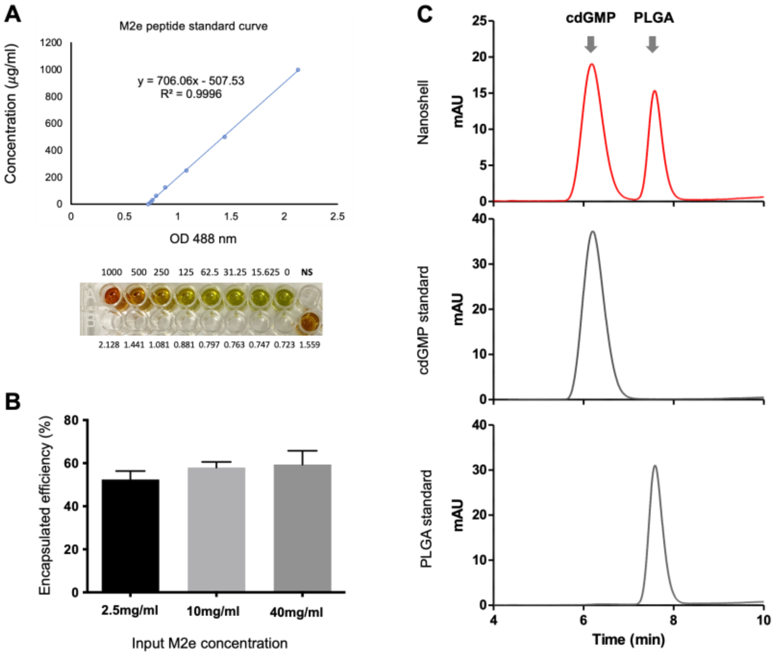

Supplementary Figure S1: Quantification of M2e peptide and cdGMP encapsulation in nanoshells by Micro BCA assay and HPLC. (A) Standard curve and a representative image for M2e peptide quantification using Micro BCA Protein Quantification Assay. (B) M2e peptide encapsulation efficiency in nanoshells prepared with inner aqueous phases containing varying concentrations of M2e peptides. (C) Quantification of cyclic di-GMP encapsulated in nanoshells by HPLC.

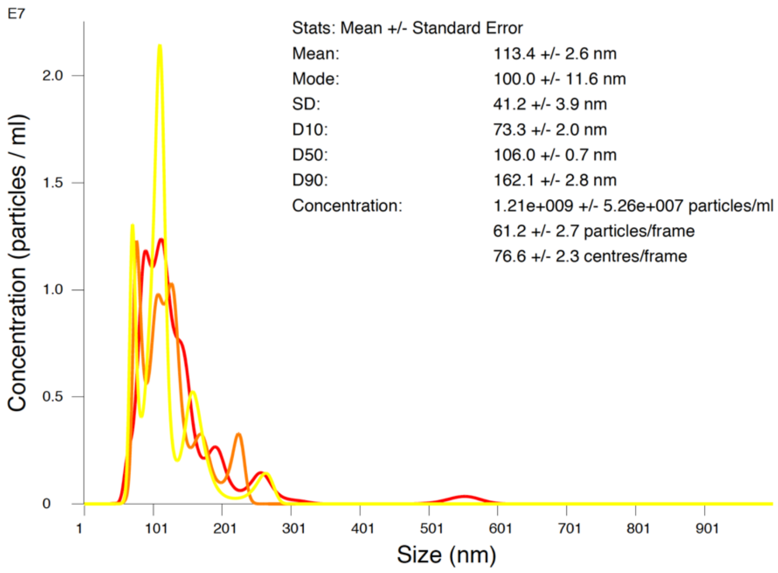

Supplementary Figure S2: Nanoshell enumeration by nanoparticle tracking analysis. For a sample containing 15 µg/mL of nanoparticles, NTA shows approximately 1.2×10^9 nanoparticles. The number translates to approximately 8×10^{11} nanoshells per 1 mg of PLGA.

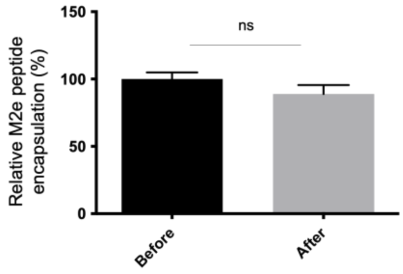

Supplementary Figure S3: M2e peptide encapsulation in the nanoshells before and after nanoshell lyophilization. The nanoshells retained their encapsulants and had negligible peptide loss following lyophilization and reconstitution.

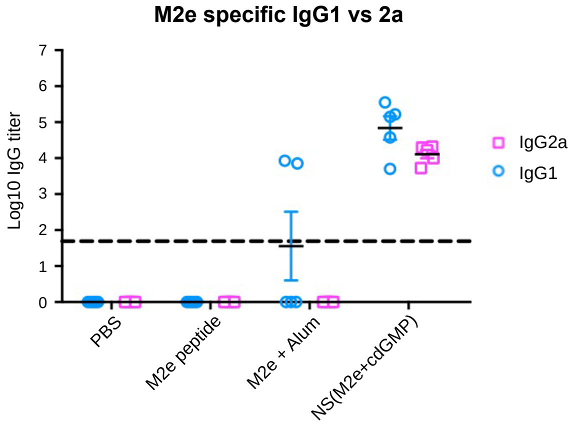

Supplementary Figure S4: M2e-specific IgG1 and IgG2a titers following a single shot immunization with PBS, M2e peptide, Alum-adjuvanted M2e peptides, and M2e nanoshell vaccine in mice on day 35 post-vaccinaiton. Error bars represent mean ± SEM (N = 5).

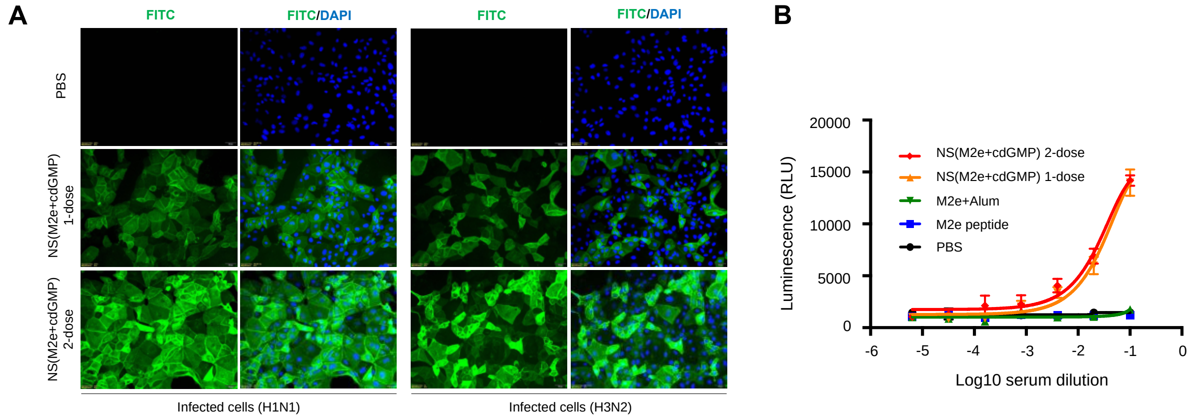

Supplementary Figure S5: Serum titer binding to influenza virus-infected MDCK cells and serum ADCC activity for mice inoculated with either one- or two-dose M2e nanoshell vaccine. (A) Immunofluorescence assay shows that the serum of mice receiving a 2-dose nanoshell vaccination regimen yielded higher antibody binding and immunofluorescence to both H1N1- and H3N2-infected MDCK cells as compared to the serum of mice receiving a 1-dose nanoshell vaccination. (B) ADCC surrogate assay shows comparable ADCC activities between sera derived from mice with 1-dose or 2-dose nanoshell vaccination.

PBS	M2e peptide	M2e+Alum	M2e NS	HA antibody	M2 antibody
<10	<10	<10	<10	80	<10

Supplementary Figure S6: Hemagglutination inhibition assay examining neutralizing capacity of anti-M2e against influenza viruses. Anti-M2e antibodies showed no observable neutralizing capacity against the influenza pathogen.

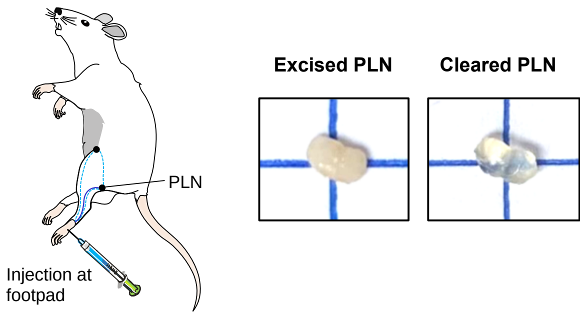

Supplementary Figure S7: Image of transparent lymph nodes following X-clarity treatment. Popliteal lymph node was excised and underwent tissue clearing treatment for tracking of M2e peptide distribution.

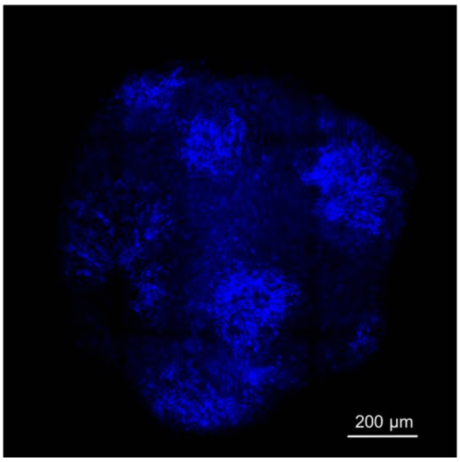

Supplementary Figure S8: Fluorescence image of draining lymph node following mice inoculation with Alexa Fluorophore 647-conjugated M2e peptides. The image was acquired 4 hours following footpad injection with fluorescently labelled M2e peptides.

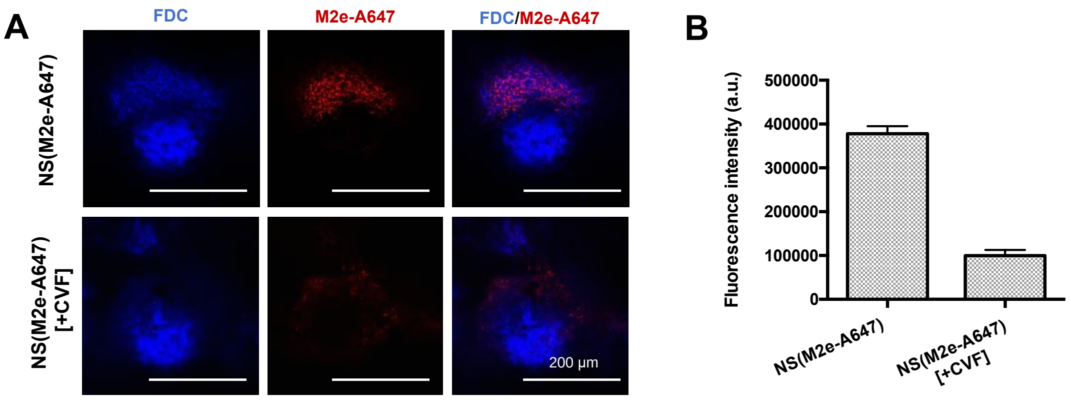

Supplementary Figure S9: Comparison of M2e antigen retention in mice lymph node follicles following M2e nanoshell inoculation. For complement factor depletion, mice received intravenous injection of cobra venom factor (CVF) 3 hours prior to nanoshell injection in the footpad. The draining lymph nodes were then excised for assessment on Day 3. (A) Representative images of lymph node follicles showing reduced M2e-A647 retention at the lymph node follicles in CVF-treated mice. (B) Quantification of M2e-A647 retention in control and CVF-treated mice. N=3.

	Size(nm)	Zeta potential	M2e EE (%)	cdGMP EE (%)
NS(M2e+cdGMP)	98.3	-46.2	56.3%	52.5%
NS(M2e+CpG)	99.7	-48.4	57.9%	50.7%
NS(M2e)	97.9	-44.6	55.9%	-

Supplementary Table S1: physicochemical properties of nanoshells encapsulation M2e antigen only, M2e + CpG-ODN 1826, and M2e + cdGMP. DLS characterization, and peptide and CpG-ODN quantification showed comparable morphology, physicochemical properties, and encapsulation efficiencies for the three different nanoshells.

	Size(nm)	Zeta potential	M2e EE (%)	cdGMP EE (%)
M2e NS	93.32	-41.1	56.3%	52.5%
M2e PEG-NS	121.0	-24.9	54.7%	51.3%

Supplementary Table S2: Physicochemical properties and encapsulated contents of PEG-free and PEG-coated M2e nanoshells.

Sequence	Origin
**SLLTEVETPIRNEWGCRCNDSSD**	**Consensus (Vaccine)**
SLLTEVETPIRNEWGCRCNGSSD	A/Puerto Rico/8/1934 (H1N1)
SLLTEVETPIRNEWGCRCNDSSD	A/Aichi/2/1968 (H3N2)
SLLTEVETPTRSEWECRCSDSSD	A/California/7/2009 (pdmH1N1)

Supplementary Table S3: M2e peptide sequences for the consensus M2e antigen for vaccine development, A/Puerto Rico/8/1934, A/Aichi/2/1968, and A/California/7/2009.
